# Towards compatibility of EUnetHTA JCA methodology and German HTA: a systematic comparison and recommendations from an industry perspective

**DOI:** 10.1007/s10198-021-01400-2

**Published:** 2021-11-12

**Authors:** Agnes Kisser, Joschua Knieriemen, Annette Fasan, Karolin Eberle, Sara Hogger, Sebastian Werner, Tina Taube, Andrej Rasch

**Affiliations:** 1 Pfizer Pharma GmbH, Linkstraße 10, 10785 Berlin, Germany; 2 AbbVie Deutschland GmbH & Co. KG, Mainzer Straße 81, 65189 Wiesbaden, Germany; 3 AMS Advanced Medical Services GmbH, Rosa-Bavarese-Str. 5, 80639 Munich, Germany; 4 Verband Forschender Arzneimittelhersteller e.V., Hausvogteiplatz 13, 10117 Berlin, Germany

**Keywords:** Act on the Reform of the Market for Medicinal Products (AMNOG), EUnetHTA, Relative effectiveness assessment, Health technology assessment, I10, I18

## Abstract

**Objective:**

The transferability of the EU joint clinical assessment (JCA) reports for pharmaceuticals for the German benefit assessment was evaluated by systematically comparing EU JCA and German clinical assessments (CA) based on established assessment elements for HTA and assessing the potential impact of differences on Federal Joint Committee (Gemeinsamer Bundesausschuss, G-BA) ability to derive the therapeutic added value.

**Methods:**

Identification of all pharmaceuticals undergoing both, EU JCA and German CA between January 2016–June 2020. Qualitative review and data extraction from the assessments, assessment of methodological differences using a hierarchical model. Recommendations for harmonisation were developed and consented with pharmaceutical industry stakeholders.

**Results:**

Differences with potentially major impact: (1) View on differing treatment algorithms and definition of corresponding subpopulations/respective comparators. (2) Clinical relevance of surrogate/intermediate endpoints. Inclusion of different/surrogate morbidity endpoints resulting in different relative effectiveness conclusions. (3) Tolerance of study interventions not used according to marketing authorisation. (4) Different operationalisation and/or weighting of individual safety endpoints leading to differing relative safety conclusions. Differences with potentially minor impact: (1) Disagreement in risk of bias assessment for overall survival and its robustness against study limitations. (2) Use of patient-reported outcome symptom scales as measurements for health-related quality of life instruments.

**Conclusion:**

While many synergies between EU JCA and German CA exist, we identified several aspects in HTA methodology that would benefit of harmonisation and ensure the transferability of future EU JCA to the German HTA process without duplicated evaluation requirements. For those, a set of recommendations was developed.

**Supplementary Information:**

The online version contains supplementary material available at 10.1007/s10198-021-01400-2.

## Introduction

Over the past decades, Health Technology Assessments (HTAs) for new medicines have become a standard feature in many European countries as part of their reimbursement decision-making processes [1, 2]. In Germany, the law reforming the pharmaceutical market (Arzneimittelmarkt-Neuordnungsgesetz—[AMNOG]), introduced in 2011, requires the Federal Joint Committee (Gemeinsamer Bundesausschuss, G-BA) to perform a comprehensive assessment of the added therapeutic value of pharmaceuticals as a basis for reimbursement price negotiations. The G-BA`s rating on added therapeutic value takes into account clinical assessments (CA) provided by the Institute for Quality and Efficiency in Health Care (IQWiG) or, for medicines with orphan designation, by the G-BA itself (designated “German CA” in the following).

Differences in HTA methodologies across European countries are well known [2–5] and may lead to delayed and unequal access by patients to medicines in Europe [6, 7]. Since 2006 and until 2021, the European network for Health Technology Assessment (EUnetHTA) developed and piloted methods and processes for cross-border collaboration on HTA in Europe, including > 20 joint clinical assessments (JCA) for pharmaceuticals and > 30 for medical devices, on a EU-funded project basis [8–11].

In 2018, the European Commission (EC) proposed a regulation to establish a sustainable model of cooperation of EU Member States on HTA [12]. The European Commission, Council and the Parliament have very recently (June 2021) reached a compromise on the EU HTA Regulation, expected to come into force in 2024. [13, 14]. The conduct of JCA is one of the main pillars of the future joint work and participation in the JCA will be mandatory for manufacturers once the regulation comes into force. A main objective of the regulation is to establish common rules and methodologies for JCA, to further promote convergence and to reduce duplication of submissions across the EU [12, 15].

To effectively reduce duplication, the results of the European JCA (designated “EU JCA” in the following) should be fit-for-use for subsequent national decisions on overall value of the technology. They should substitute national CA with ideally no complementary clinical analyses needed at Member States level unless justified by the specific national health care context [16–18]. The clinical evidence should be assessed at Union-level in a consistent way, according to established methods and criteria, irrespective of the national HTA body, which is appointed to carry out the EU JCA. The aim of this analysis is to evaluate the transferability of the current EU JCA for pharmaceuticals into the German HTA process.

During the last years, several analyses evaluated guidelines, methods and outcomes of HTAs on national versus European level, focusing on selected countries, indications or on specific products [1, 4, 19–22]. To our knowledge, the question of transferability of EU JCA to German CA has so far not been investigated in detail. Three analyses focused on the HTA Core Model as basis for JCA which was considered as useful and flexible framework for standardized evidence generation [19, 20, 22]. Three publications compared EU JCA to national reports [2, 4, 21]. Thereby, two analyses focused on specific products (pazopanib [1] or alectinib, midostaurin and regorafenib [21]) in selected countries. These revealed that EU and national assessments share methodological elements, e.g., main comparators and outcomes, but also explored heterogeneity in the interpretation of evidence due to the different relevance of indirect comparisons or use of endpoints. However, the analysis of Kleijnen et al. [1] is based on one JCA carried out during EUnetHTA Joint Action (JA)1 (2010–2012) and reflects the then current state of EUnetHTA methodology, which has evolved since JA1. The comparison also does not include German CAs. The comparison by Jose et al. [21] includes assessments from EUnetHTA JA1 to JA3 and the respective German CA. As it is published as abstract only, there is not enough detailed information to evaluate the impact of differences seen between the EU JCA and German CA and to answer our question of interest. The comprehensive analysis by Chassagnol et al. [4] includes 12 JCA from JA1 to JA3 and national assessments of France, Italy, Germany and UK. Since the comparison comprises four countries, methodological aspects are reported rather high level and provided information is considered as not sufficiently detailed to answer our question of interest.

For our analysis, we systematically compared EU JCA and German CA based on established assessment elements of HTA [23, 24] and assessed the potential impact of differences on G-BA’s ability to decide on the therapeutic added value. We chose a hierarchical approach using the decision-points during any CA allowing us to rank differences in methodological approaches based on their timing within an assessment and the subsequent impact on the assessment content and results.

The systematic comparison of EU JCA and in particular German CA based on a hierarchical approach distinguishes our analysis from previously published comparisons of EU JCA versus national assessments.

## Methods

**Fig. 1:**
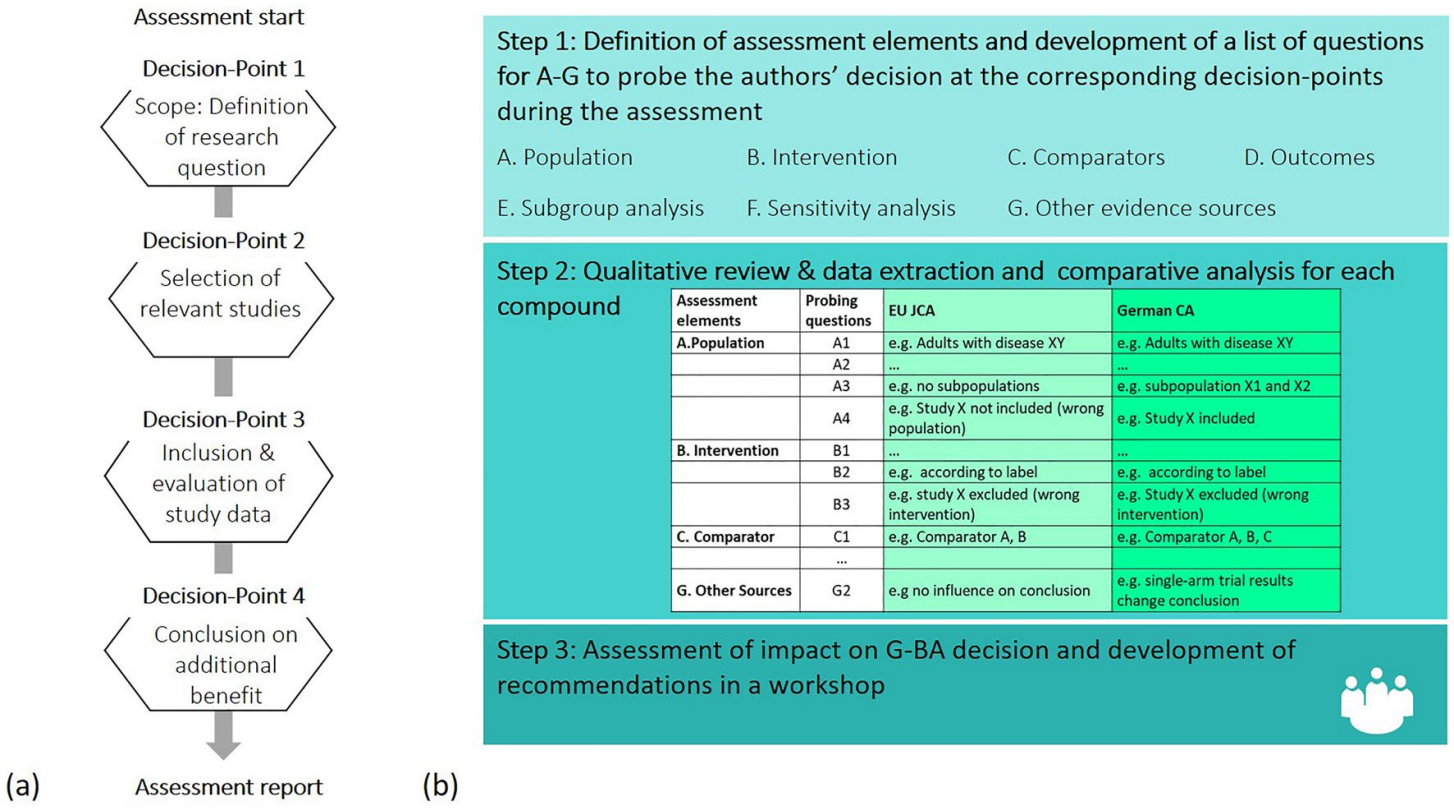
(**a**) The four decision-points during a clinical assessment. (**b**) The hierarchical approach of the analysis

All pharmaceutical compounds that had undergone both EU JCA (during EUnetHTA JA 3) and German CA between January 2016 and June 2020 were identified and systematically compared. Only JA3 EU JCA were included in the analysis to reflect the most current state of EUnetHTA methodology, which has evolved over the three JA. Data were obtained from the relative clinical effectiveness assessments conducted and published by EUnetHTA [25–29] and G-BA/IQWiG [30–34]. The underlying assumption is that future EU JCA should substitute national CA, while the final decision on added therapeutic value remains in the responsibility of the national decision maker—in Germany: the G-BA. The G-BA may—and often does—deviate in its decision from the CA`s conclusion. Therefore, our comparison is based on the CA reports and not on the final decisions on additional benefit. Details on indication, posology and administration of each product were derived from the summaries of product characteristics (SmPCs) available on the European Medicines Agency (EMA) website [35–39].The analysis followed three steps (Fig. 1b).

**Step 1:** Based on the PICO scheme and established methodology for HTA [23, 24], we specified the following assessment elements for comparison of methodology: (A) population, (B) interventions, (C) comparators and (D) the outcome categories mortality, morbidity, health-related quality of life and safety, (E) subgroup analyses, (F) sensitivity analyses and (G) other sources of evidence.

During any CA, there are four decision-points: (1) definition of the research question, (2) selection of the studies relevant for the assessment, (3) evaluation of the study data, (4) derivation of a conclusion on relative effectiveness and safety (Fig. 1a). Depending on the decision-point, different decisions vary in their effects on the subsequent content and the conclusion of the assessment. A set of questions was specified for each assessment element (A–G) to probe the authors` decision at the corresponding decision-points during the assessment process. A detailed listing is shown in Supplementary Table 1.

**Step 2:** For each compound, a qualitative review of the assessments was performed and data were extracted from the EU JCA and German CA for each of the assessment elements (A-G). Extracted data were reviewed by two independent reviewers and deviations were resolved by discussion. Based on the data extraction, methodological differences between EU JCA and German CA in use and interpretation of the evidence were identified and analysed with regards to their effect on the content and conclusions of the assessment.

**Step 3:** During a workshop, representatives from Abbvie, AstraZeneca and Pfizer as members of the Local Area Working Group (LAWG), representatives of the German Association of Researching Pharmaceutical Manufacturers (vfa), representatives of the two marketing authorisation holders of the products included in the analysis (Novartis and Roche), and HTA experts (*AMS* Advanced Medical Services GmbH) assessed the potential impact of methodological differences identified in Step 2 on the G-BA’s ability to derive a decision on added therapeutic value.

The data extraction table (Step 2), a slide set summarising synergies and differences for every value domain or topic (A-G) and a pre-read document outlining the project objectives and methods were shared before with all 12 participants. The discussion was led by a moderator and documented by a minute-taker.

In view of a transferability of EU JCA into the German HTA, differences due to evaluation of additional data, comparators, etc., that did not affect the conclusion of the assessments were deemed to have no impact. Differences with major impact were defined as either relevant information missing in EU JCA for German CA (data on comparator, relevant endpoints, etc.), or leading to different conclusions on relative effectiveness/safety. All other methodological differences were classified as of minor impact. As EU JCA do not provide an overall recommendation/conclusion on added value across endpoints and comparators, conclusions were compared within the endpoint categories (mortality, morbidity, health-related quality of life [HRQoL] and Safety) and for each comparator separately. The comparison is found in Supplementary Table 2.

Based on the impact assessment, the workshop participants in coordination with representatives of vfa and LAWG developed recommendations for harmonisation of methodologies. Recommendations were subsequently consented with the vfa Subcommittee for Benefit Assessment and the LAWG Team for HTA.

## Results

Until June 2020, five products have undergone both the EU JCA process in the framework of EUnetHTA's JA3 and the German CA procedure (AMNOG). Of these, midostaurin was the first (November 2017) and brolucizumab the most recent (March 2020) product assessed by EUnetHTA. All five products (see Table 1) were included in the analysis, among them two orphan drugs (midostaurin, polatuzumab). Three products were oncology products (midostaurin, polatuzumab, alectinib), two were non-oncology products (siponimod, brolucizumab).

**Table 1:** Medicines^1^ included in the analysis: comparison of relevant comparators

Relevant comparators in the (J)CAs		Reason for the difference
**Midostaurin** **EMA approval: Acute Myeloid Leukaemia FLT3 + ; Newly Diagnosed (Sep 18th 2017)**		
EU JCA publication: Nov 8th 2017 (co-) author country: Finland (Norway)	German CA publication: Jan 15th 2018 author: G-BA	
Standard induction and consolidation chemotherapy (cytarabine in combination with daunorubicin 60 mg/m^2^/day during the induction phase) Induction and consolidation chemotherapy, using daunorubicin 90 mg/m^2^/day (instead of 60 mg/m^2^/day) during the induction phase	Midostaurin with standard induction and consolidation	The German CA for orphan drugs is based on the registration trials only. No definition of additional comparators
**Brolucizumab** **EMA approval: Neovascular (wet) age-related macular degeneration (Feb 13th 2020)**		
EU JCA publication: Mar 12th 2020 (co-) author country: Finland (Spain)	German CA publication: Jul 28th 2020 author: IQWiG	
Aflibercept 2 mg/0.05 ml Ranibizumab 0.5 mg/0.05 ml Bevacizumab 1.25 mg/0.05 ml	Ranibizumab Aflibercept	EU JCA included off-label comparator bevacizumab due to its relevance in several European healthcare systems
**Siponimod** **EMA approval: Secondary progressive multiple sclerosis (SPMS) with active disease evidenced by relapses or imaging features of inflammatory activity (Jan 13th 2020)**		
EU JCA publication: Mar 3rd 2020 (co-) author country: Portugal (Ireland)	German CA publication: May 13th 2020 author: IQWiG	
Interferon-β-1a or -β-1b plus BSC Mitoxantrone plus BSC Ocrelizumab plus BSC Natalizumab plus BSC Fingolimod plus BSC Cladribine plus BSC Rituximab plus BSC	a. Interferon-beta 1a or interferon-beta 1b or ocrelizumab^2^ b. BSC^3^	As the patient population was split in the German CA in two subpopulations, two comparators are defined according to these populations. EUnetHTA defined seven active comparators for the whole population
**Polatuzumab** **EMA approval: Relapsed/refractory diffuse large B-cell lymphoma (DLBCL) who are not candidates for haematopoietic stem cell transplant (Jan 16**^**th**^** 2020)**		
EU JCA publication: Feb 13th 2020 (co-) author country: Germany (France)	German CA publication: May 15th 2020 author country: G-BA	
*PICO 1a* Antineoplastic therapy according to physician’s choice under consideration of the previous therapy and patients characteristics, can include: Platinum- and/or gemcitabine-based regimens (like GemOx) Platinum-based regimens (like ICE or DHAP ± R) without conditioning chemotherapy for transplant (if necessary with reduced dosage) Rituximab bendamustine combination^4^ *PICO 1b* After failure of two or more therapies the comparator can include: Axicabtagene ciloleucel Tisagenlecleucel Pixantrone Rituximab–bendamustine combination^4^ BSC	Rituximab-bendamustine	The German CA for orphan drugs is based on the registration trials only. No definition of additional comparators
**Alectinib** **EMA approval: First-line treatment of adult patients with ALK + advanced non-small-cell lung cancer (NSCLC) (Dec 18th 2017)**		
EU JCA publication: Feb 23rd 2018 (co-) author country: Sweden (Austria/ Croatia)	German CA publication: Mar 28th 2018 author country: IQWiG	
Crizotinib Ceritinib	Crizotinib	According to the four criteria for comparator derivation, the G-BA prefers the comparator with additional benefit (crizotinib). The German CA of ceritinib did not prove an additional benefit, therefore ceritinib is not included as comparator

*BSC* best supportive care, *CA* clinical assessment, *JCA* joint clinical assessment, *PICO* patient, intervention, comparator and outcome

^1^Data were obtained from the relative clinical effectiveness assessments conducted and published by EUnetHTA (https://eunethta.eu/) and G-BA/IQWiG (https://www.g-ba.de/). Details on indication, posology and administration of each product were derived from the SmPCs available on the European Medicines Agency (EMA) website (https://www.ema.europa.eu/en)

^2^Patient population: Adult patients with secondary progressive multiple sclerosis (SPMS) with active disease, defined by clinical findings or imaging of the inflammatory activity, with relapses

^3^Patient population: Adult patients with secondary progressive multiple sclerosis (SPMS) with active disease, defined by clinical findings or imaging of the inflammatory activity, without relapses

^4^Specifically in elderly patients or patients with comorbidities

### Population (A)

For all products in both EU JCA and German CA, the population in scope of the assessment corresponded to the approved indication according to SmPC (questions A1 and A2 in Supplementary Table 1). No differing decisions on suitability of the study populations during study pool selection were identified (A4).

For two products, the label population was split into subpopulations based on differing therapy situations, revealing in both cases differing decisions of the EU JCA and the German CA authors (A3). In the polatuzumab assessment, the EU JCA authors defined subpopulations within and in addition to the overall label population according to their failure on previous treatment options, requiring additional analyses of the corresponding study subpopulations. These subpopulations were not in the scope of the German CA. Conversely, in the siponimod assessment, no subpopulations were specified for the EU JCA, whereas the German CA differentiated two subpopulations with varying therapeutic goals according to the presence or absence of relapses. These differences in assessment scope have a major effect on the content of the report, as they affect all subsequent steps in the assessment. They may have a major impact on the transferability of the EU JCA, if—as in the latter case—relevant information for German decision-making would be omitted in the EU JCA.

### Intervention (B)

In general, the intervention in scope was defined according to the corresponding EU marketing authorisation (questions B1 and B2 in Supplementary Table 1).

Our analysis revealed that some deviations in the pharmaceutical form from the marketing authorisation were tolerated in both EU JCA and German CA (B3). Although the liquid formulation of polatuzumab used in the pivotal study deviated from the finally authorised lyophilised formulation, the assessment authors regarded the application of the intervention as suitable and included the study in both assessments. Overall, no differing decisions during study pool selection with regards to the intervention were identified.

### Comparator (C)

The comparators in scope of the EU JCA and German CA (question C1 in Supplementary Table 1) are presented in Table 1. For all products, the EU JCA authors defined additional comparators to the relevant comparators of the corresponding German CA. The differences in the selection of comparators for each product were due to various reasons summarised in Table 1. For 4/5 products, all relevant comparators of the corresponding German CA were included in the EU JCA. For one product, however (siponimod), German CA authors identified a subpopulation (Secondary Progressive Multiple Sclerosis without relapses) for which no approved medicinal products were available and consequently defined best supportive care (BSC) as appropriate comparator for this subpopulation. Again, as differences appeared already in the scope of the assessment, they had major effects on the content of the assessment: available data for a direct comparison with BSC were omitted and not assessed in the EU JCA, which would thereby not have been transferable for German decision-making as relevant research questions were not addressed.

Randomized controlled trials (RCT) with relevant comparators for both EU JCA and German CA were available for four products (C2). For three products, the EU JCA and German CA authors agreed on the inclusion of direct comparative studies against at least one common comparator. In the broluzicumab assessments, there was disagreement regarding tolerable deviations of the comparator aflibercept from the dosing scheme according to its current SmPC (C3). This led to exclusion of the two pivotal RCTs against aflibercept from the German CA whereas the EU JCA authors included the study results in their assessment. This difference was deemed as having no impact on transferability, as the EU JCA contained all relevant information for German decision-making.

If direct comparisons were not available for one or more relevant comparators, 4/5 EU JCA considered additional indirect comparisons (C4) whereas none of the German CA included indirect comparisons. Only 2/5 EU JCA however used the results from indirect comparisons to derive a conclusion on relative effectiveness or relative safety (C5).

Due to the differing decisions during the definition of project scope and/or study selection described above, the assessments of siponimod and broluzicumab included results from different comparisons. Therefore, no evaluation of synergies and differences with regards to the evaluation of study results and conclusions on relative effectiveness and safety—described subsequently—between EU JCA and German CA was possible for these two products.

### Outcomes (D)

Outcomes were divided in the main categories: mortality (overall survival), morbidity (clinical events, symptoms, function due to disease or its treatment), health-related quality of life (HRQoL) and safety (adverse events due to disease or its treatment).

#### Mortality

Overall survival (OS) was unanimously considered in scope for four products (question D1 in Supplementary Table 1) and no deviations were identified in terms of operationalisation (Table 2). OS was not seen as a relevant endpoint for the indication of broluzicumab (macular degeneration) in the EU JCA as opposed to the German CA.

**Table 2:** Comparison of relevant endpoints according to project scope

Relevant endpoints in the (J)CAs		Reason for the difference
***Midostaurin***		
***EU-JCA***	***German CA***^***1***^	Different morbidity endpoints defined as relevant (indicated in **bold**) As the G-BA does not consider the morbidity endpoints to be relevant for the added benefit, but presents them in part as supplementary, there is no missing information relevant to the assessment in the EU-JCA
Mortality Overall survival	Mortality Overall survival	
Morbidity Disease free survival Complete remission **Event-free survival** **Cumulative incidence of relapse** **Proportion of patients who discontinued the treatment**	Morbidity Disease free survival Complete remission **Stem cell transplantation**	
HRQoL Not evaluated in the relevant trial	HRQoL Not evaluated in the relevant trial	
Safety Any adverse events (AEs) Serious AEs (SAEs) Grade ≥ 3 AEs Discontinuation due to AE Death as SAE AE of special interest	Safety Any AEs SAEs Grade ≥ 3 AEs, Discontinuation due to AE Death as SAE AE of special interest	
***Brolucizumab***		
***EU-JCA***	***German CA***	For German CA, no relevant studies were available, as IQWiG considered the comparator therapy not adequately addressed (ranibizumab and aflibercept dosing was not in line with SmPC). Thus, endpoints were not assessed in the German CA
Mortality –	Mortality –	
Morbidity **Best corrected visual acuity (treated eye)** **Anatomic parameters of disease activity**	Morbidity –	
HRQoL **Vision-related quality of life (NEI VFQ-25 instrument)**	HRQoL –	
Safety **Any AEs** **SAEs** **Discontinuation due to AE** **Death as a serious AE** **AE of special interest (e.g., ocular/non-ocular AEs)**	Safety –	
***Siponimod***		
***EU-JCA***	***German CA***	Different morbidity/HrQoL endpoints defined as relevant (indicated in **bold**). Patient reported outcome (PRO) data were assigned to different outcome-domains. While the symptom scales EQ5D-VAS and MSIS-29 (physical and mental function scale) were evaluated as morbidity outcomes in the German CA, EU-JCA treated these scales as health related quality of life (HRQoL) outcomes
Mortality OS	Mortality OS	
Morbidity Confirmed disability progression (EDSS-based); time to CPD/6-month, **time to CPD/3 month; proportion with CPD** Cognitive function (assessed using the Symbol Digit Modalities Test [SDMT] and the Brief Visuospatial Memory Test—Revised [BVMT-R]) Symptoms (e.g., fatigue **and cognitive, bowel, and bladder dysfunction**)	Morbidity Confirmed disability progression (EDSS-based, confirmed over a 6-month period) Cognitive function (assessed using the SDMT and the BVMT-R) Fatigue	
Walking ability (assessed using the Multiple Sclerosis Walking Scale-12 [MSWS-12]) Clinical relapse (e.g., annualised relapse rate, proportion of relapse-free patients based on the Expanded Disability Status Scale (EDSS)) Timed 25-Foot Walk Test (T25FW) Nine-Hole Peg Test **Rate of patients that become confined to wheelchair use** Progression of Paced Auditory Serial Addition Test (PASAT) **MRI-measured inflammatory disease activity and burden (T1 gadolinium-enhancing lesions, new or enlarging T2 lesions, brain volume)** **No evidence of disease activity (NEDA; absence of progression, relapses, and MRI activity)**	Walking ability (MSWS-12) Confirmed disease relapses (EDSS-based) **Visual acuity (assessed using the Low Contrast Visual Acuity [LCVA])** Severity of disability (assessed using the Multiple Sclerosis Functional Composite [MSFC]), including Timed 25-Foot Walk Test (T25FW) Nine-Hole Peg Test Progression of Paced Auditory Serial Addition Test (PASAT) **Physical function (assessed using the MSIS-29, physical function scales)** **Mental function (assessed using the MSIS-29, mental function scales)** **Health status (assessed using the VAS of the EQ-5D)**	
HRQoL **Physical function (assessed using the Multiple Sclerosis Impact** **Scale-29 [MSIS-29], physical function scales)** **Mental function (assessed using the MSIS-29, mental function scales)** **Health status (assessed using the visual analogue scale [VAS] of the European Quality of Life Questionnaire 5-Dimensions [EQ-5D])**	HRQoL **Health related Quality of Life**	
Safety SAEs Discontinuation due to AE **AE** **Treatment related mortality**	Safety SAEs Discontinuation due to AE **AE of special interest (including infections and bradycardia)**	
***Polatuzumab***		
***EU-JCA***	***German CA***^***1***^	Different morbidity and safety endpoints defined as relevant (indicated in bold)
Mortality Overall survival	Mortality Overall survival	
Morbidity Complete response Symptoms (e.g., like B-symptoms or fatigue) **Progression free survival (PFS)**	Morbidity Complete Response Symptoms (e.g., like B-symptoms or fatigue)	
HRQoL Health related Quality of Life	HRQoL Health related Quality of Life	
Safety AE SAE AE according to CTCAE AE resulting in withdrawal (overall and by SOCs and PT, respectively)	Safety AE, SAE AE according to CTCAE, AE resulting in withdrawal (overall and by SOCs and PT, respectively) **Prespecified AESI**	
***Alectinib***		
***EU-JCA***	***German CA***	Different morbidity endpoints defined as relevant (indicated in bold)
Mortality Overall survival	Mortality Overall survial	
Morbidity **Progression free survival (PFS)** **Time to CNS progression** **Objective response rate** **CNS duration of response (DOR)**	Morbidity **Health status (EQ-5D-VAS)** **Symptoms measured by the symptom scales of the instruments European Organization for Research and Treatment of Cancer (EORTC) QLQ-C30 und Quality of Life Questionnaire Lung Cancer 13 (QLQ-LC13)**	
HRQoL **Symptom scales of QLQ-C30** **Quality of Life Questionnaire Lung Cancer 13 (QLQ-LC13)**	HRQoL Functional scales of the EORTC QLQ-C30	
Safety SAEs SAEs CTCAE grade ≥ 3 Discontinuation due to AEs, AE of special interest/ **Any AEs** **Severe AEs** **Death as SAE** **AE leading to dose reduction**	Safety SAEs SAEs CTCAE grade ≥ 3 Discontinuation due to AEs Other specific AEs, if applicable	

*AE* adverse event, *AESI* adverse events of special interest, *BVMT* Brief Visuospatial Memory Test, *CA* clinical assessment, *CDP* confirmed disability progression, *CNS* central nervous system, *CTCAE* Common Terminology Criteria for Adverse Events, *DOR* duration of response, *EDSS* Expanded Disability Status Scale, *EORTC* European Organization for Research and Treatment of Cancer, *EQ-5D* Quality of Life Questionnaire 5-Dimensions, *HRQoL* health related quality of life, *JCA* joint clinical assessment, *LCVA* Low Contrast Visual Acuity, *MRI* magnetic resonance imaging, *MSFC* Multiple Sclerosis Functional Composite, *MSIS-29* Multiple Sclerosis Impact Scale-29, *MSWS-12* Multiple Sclerosis Walking Scale-12, *NEDA* no evidence of disease activity, *NEI* National Eye Institute, *OS* overall survival, *PASAT* Paced Auditory Serial Addition Test, *PFS* progression free survival, *PT* preferred term, *QLQ-C30* Quality of Life Questionnaire, *QLQ-LC13* Quality of Life Questionnaire Lung Cancer 13, *SAE* Serious AE, *SDMT* Symbol Digit Modalities Test, *SOCs* system organ class, *SmPC* summary of product characteristics, *T25FW* Timed 25-Foot Walk Test, *VAS* visual analogue scale, *VFQ* vision-related quality of life

^1^Orphan drug designation: The additional benefit is considered proven with the approval (up to a turnover limit of € 50 million during the last 12 month). The G-BA only decides on the extent of the benefit

We found no disagreement in deriving conclusions on relative effectiveness for OS (D2), but differing estimations were made in risk of bias (RoB) assessment (D3) and grading of the quality of evidence (D4) in the polatuzumab and alectinib assessments. In both cases, the authors of the EU JCA regarded OS as less robust against limitations in the study design (e.g., lack of hypothesis testing) compared to AMNOG procedures. In the polatuzumab EU JCA, the certainty of evidence for OS was downgraded, as the protocol did not contain any hypothesis of superiority or non-inferiority and no detailed prespecified statistical analysis plan (SAP) was available. Thus, it was unclear for the EU JCA authors whether reporting of the outcome was independent of the results. In the alectinib EU JCA, the certainty of evidence was downgraded for OS as the study was not powered for OS superiority, patients were treated at the discretion of the investigator after disease progression and only results from an interim analysis were available.

#### Morbidity and HRQoL

For none of the five products analysed, the same set of morbidity endpoints was defined in the scope of German and EU JCA (question D1 in Supplementary Table 1).

EU JCA authors generally included broader sets of morbidity endpoints in their assessments (Table 2). Our analysis revealed differing views on the relevance of surrogate and intermediate outcomes based on laboratory measurements or imaging, such as complete response (CR), progression-free survival (PFS) or event-free survival (EFS) as intermediate endpoints for OS or cumulative incidence of relapse (CIR) and patients who discontinued treatment as supportive endpoints.

Generic and disease-specific HRQoL endpoints were in scope of all assessments. Whereas in German assessments symptom scales of PRO like QLQ-C30 & QLQ-LC13, EQ5D-VAS or MSIS-29 were evaluated as morbidity outcomes, authors of EU JCA treated these scales as HRQoL outcomes.

A juxtaposition of all clinical endpoints considered in the EU JCA and German CA is presented in Table 2.

Differing sets of morbidity endpoints also led to disagreement in deriving conclusions on relative effectiveness for morbidity (D2). Differing estimations were also made in risk of bias (RoB) assessment (D3) and grading of the quality of evidence (D4), which resulted in a major effect. For alectinib and polatuzumab, the EU JCA authors drew a positive conclusion in the category morbidity whereas the German CA authors found no evidence for an advantage in this category.

For midostaurin, the EU JCA found a low RoB for the endpoint disease-free survival (DFS) whereas the German CA considered the RoB as high due to non-randomization. For alectinib, the EU JCA authors assigned low RoB and high quality of evidence for all endpoints, whereas the German CA authors determined high RoB primarily due to high missing rates (> 30%) and downgraded the quality of evidence due to high RoB and only one available head-to-head trial.

#### Safety

Adverse events (AE), serious AE, severe AE, AE leading to therapy discontinuation and pre-specified AE of special interest (AESI) were consistently considered as in scope (question D1 in Supplementary Table 1) of both, EU JCA and German CA (Table 2). Some differences with no effect were identified regarding the specification of endpoints in the category of safety (prespecified AESI, AE of particular relevance) in the German CA for siponimod, midostaurin and polatuzumab.

Another difference was identified regarding the operationalisation of safety endpoints. For polatuzumab, the German CA authors used observation-time adjusted effect estimates for safety endpoints in contrast to the EU JCA, where no adjustment for different observation periods was included. This had a major effect on the conclusion on relative safety (D2): German CA authors derived an unfavourable balance based on AE leading to discontinuation and in individual AE of special interest, whereas the EU JCA authors found no difference in safety outcomes between polatuzumab and the comparator. The impact of these methodological differences was therefore rated as major.

There was overall concordance in the RoB assessment (D3) and evaluation of quality of evidence (D4) of the safety endpoints: for polatuzumab and alectinib, both EU JCA and German CA authors found a high RoB and downgraded the quality of evidence due to open label study design. For midostaurin, no RoB was conducted in the EU JCA, the German CA authors considered the RoB as low, in both assessments quality of evidence was not downgraded.

### Subgroup analysis (E)

Subgroup analyses can be conducted to assess the generalisability of trial results by demographic (e.g., age, gender, weight), treatment history (stage of disease, prior treatment, etc.) or other parameters. The analysis identified little variation in the subgroups in scope for the assessments, typically more subgroup analyses for more endpoints are included in the German CA (E1). However, in none of the assessments included in our analysis, the subgroup analyses changed the conclusion on relative effectiveness based on the main study population (E2). Therefore the impact of this difference on transferability was deemed low.

### Sensitivity analysis (F)

Sensitivity analyses are used to assess the robustness of the findings or conclusions based on primary analyses of data in clinical trials. They allow to assess the impact, effect or influence of key assumptions or variations—such as different methods of analysis, definitions of outcomes, protocol deviations, missing data, and outliers—on the overall conclusions of a study [40]. Additional sensitivity analyses were included in several of the German CA such as sensitivity analysis of the endpoint EQ-5D-VAS (deterioration by 10 points) or sensitivity analyses for additional endpoints after censoring patients who received stem cell transplantation (F1). However, in our data set, none of the sensitivity analyses were found to change the conclusion based on the primary analysis of the outcome in the EU JCA or the German CA (F2). Therefore the impact of these differences on transferability was deemed low.

### Other sources of evidence (G)

The comparison showed that overall EU JCA include more data from other sources of evidence (G1). Single-arm studies were included in three EU JCA to complement the assessment, e.g., on specific patient groups, other formulation of the intervention, or on adverse events. Conversely, only for one product (polatuzumab), data from a single-arm study were also included as complementary evidence in the German CA.

However, in our data set, none of the other sources of evidence were found to change the conclusion based on the pivotal studies in the EU JCA or the German CA (G2). Therefore, the impact of these differences on transferability was deemed low.

## Discussion

The previous comparison of Chassagnol et al. of EU JCA and national CAs with different focuses found, that EU JCA has a more inclusive approach to evidence than the German CA [4]. The analysis revealed a high heterogeneity across the HTA appraisals of the four countries (France, Italy, Germany and UK). They concluded that compared to the German CA, the EU JCA, especially the more recent JA3 assessments, had a more inclusive approach with regards to available evidence. Indirect treatment comparisons and single arm trials were included and considered as an evidence element [4]. At the same time, the authors stress that a standardised approach over the three JA for e.g. the choice of endpoints or subgroups has been lacking, and the respective approaches in the EU JCAs have been evolving over the assessments [4].

Our analysis included five products assessed by EUnetHTA during JA3 and their respective German CA. We identified several methodological aspects with general agreement between EU JCAs and the respective German CA. We confirmed the more inclusive approach of EU JCA with regards to indirect comparisons and single arm trials. As JCA still primarily relied on the pivotal RCT to derive their conclusions, we found this difference to have limited impact on transferability. Conversely, subgroup and sensitivity analyses were more abundantly requested/included in German CA, but none were found to lead to different conclusions compared to EU JCA in our data set; again, limiting the impact on transferability. This raises questions about the extent to which subgroup and sensitivity analyses should be provided for the EU-HTA JCA.

Also, as previously reported, EU JCA were more inclusive with regards to surrogate endpoints, in our dataset leading to differing conclusions regarding the benefit shown based on these endpoints. For methodological differences between EU JCA and German CA with potential impact on the G-BA’s ability to derive a decision on added therapeutic value based on the EU JCA alone., the working group has developed a set of recommendations, summarised in Table 3.

**Table 3:** Methodological recommendations to ensure transferability of future EU JCAs for the German benefit assessment process

Domain	Recommendation
Population	- Within the therapeutic indication the definition of subpopulations with different therapeutic situations should be based on current European evidence-based guidelines
Study Intervention	- Evidence from pivotal studies should be applicable for the assessment and used preferably. Decisions on the acceptability of possible deviations of the study interventions from the approved administration should be clarified in consultation with the regulatory authority. In case of minor deviations, a pragmatic approach is recommended
Comparator	- For comparator selection, medicines with marketing authorisation for the therapeutic indication should be given priority, off-label therapies with demonstrated clinical efficacy for the therapeutic indication should be considered. Selection should be made according to available clinical evidence and European guidelines - The PICO Survey amongst EUnetHTA partners should enable a transparent, timely and consistent process to establish a consensus on Standard of care selection amongst EU Member States and, therefore, should replace the national selection. Any decisions within the subsequent national appraisal process must remain separately, i.e., the PICO Survey national comparator must remain basis of the national appraisal process
Endpoints	- Assessment methods for endpoints should be harmonised - Prespecified clinical trial test hierarchies are not recommended in an HTA because of the different scopes of HTA and drug approval. For HTA, an evaluation is intended across multiple endpoint categories - In line with the scope of the HTA, the inclusion of surrogate endpoints accepted in the marketing authorisation as well as the consideration of patient-reported symptoms, HRQoL and adverse events is advocated to enable a patient-centered assessment in all four endpoint categories (mortality, morbidity, HRQoL and adverse events) - Differences in observation times between study arms should be accounted for in the assessment of endpoints via an adequate methodology
Subgroup Analysis	- Subgroup analyses should be considered very cautiously due to their possible exploratory character. Conclusions about differential effects in subgroups should only be drawn based on adequate statistical interaction tests and only with sufficient credibility through biological plausibility (with clinical, pharmacological, or mechanistic rationale) and replication (in multiple data sources)

*EU* European Union, *HTA* health technology assessment, *HRQoL* health-related quality of life, *JCA* joint clinical assessment, *PICO* population, intervention, comparator, outcome

### Split into subpopulations based on differing therapy situations

Standard of care (SoC), including treatment pathways, varies between EU Members States and is also influenced by prior national HTA decisions. Differing views on treatment algorithms and the definition of corresponding subpopulations and their respective comparators will also affect study selection Therefore, the definition of subpopulations with different therapeutic situations within the therapeutic indication should be based on current European evidence-based guidelines.

Currently it is proposed to solve this by consulting HTA bodies and stakeholders on the relevance of proposed patient groups, comparators and endpoints during the early scoping phase. The aim is to adopt a PICO question applicable for most European countries [41, 42]. However, a definition of several PICOs to reflect varying SoC and national needs should be counterbalanced with established treatment standard according to European marketing authorisation and therapy guidelines.

### Surrogate endpoints

Both German and EUnetHTA methods state, that surrogate endpoints can be considered—although final clinical endpoints are preferred—provided the validity of the surrogate/final clinical endpoint relationship has been previously clearly established and data on all validation steps provided [23, 43].

EU JCA are timed to be conducted in parallel to the marketing authorisation process. Evidence on a final clinical endpoint that directly measures clinical benefit might not yet be available at that stage and assessors might need to recur to surrogate endpoints to predict clinical benefit. Variation in the acceptance of surrogate markers exists across HTA bodies in Europe, which might remain without detailed advice on the adequacy of surrogate markers, the validation process and statistical methods [44]. Therefore, it is advocated that surrogate endpoints accepted in the marketing authorisation are also included to enable a patient-centered assessment.

### Tolerance on deviations of study interventions from marketing authorisation

We found differing decisions regarding tolerability of deviations of study interventions from their authorised formulation during selection of the studies eligible for the assessment. As this is an early decision point in the assessment process, differing decisions at this point might significantly affect the evidence included and evaluated in the respective assessments. In this context, evidence from pivotal studies should be applicable for the assessment and used preferably, whereby harmonisation of approaches is advisable in the event of deviations, also in consultation with the regulatory authority.

### Consideration and weighting of individual safety endpoints

For safety endpoints, our analysis showed differences in the operationalisation and/or in the weighting of individual safety endpoints in the overall safety profile leading to differing conclusions on relative safety. To allow a fair comparison, appropriate observation time adjusted analyses of safety endpoints should be used.

### Different criteria for RoB assessment and prespecified test hierarchies

Substantial disagreement was found in the RoB assessments in all endpoint categories. In particular, RoB assessment for the hard endpoint OS—despite the same study base—differed between European and German CA authors, the former found this endpoint less robust against study limitations than the latter. This finding was ranked as of minor effect, despite the disagreement in RoB assessment both EU JCA and German CA authors derived the same conclusion on relative effectiveness for this endpoint.

It is reflective of an ongoing debate on relevant domains for RoB [45] and represents a difference which cannot be solved by the definition of additional PICOs or additional analyses. HTA and regulatory decision making pursue different purposes. Regulatory bodies evaluate—and need confirmation—that a medicine is effective and has acceptable side effects. HTA agencies evaluate if medicines have added value compared to what is used in clinical practice which would justify any additional costs. In that view, it is not surprising that some authors found that market authorisation is more confirmatory than (German) early benefit assessment as it includes a higher proportion of primary endpoints. [46]. Added value can be derived also from secondary or exploratory endpoints; clinical trial test hierarchy is not necessarily reflective of the relevance of the endpoints to the patients. There should be consensus for the criteria of RoB assessment amongst HTA bodies.

### Instruments for HRQoL

Several PRO symptom scales (e.g., QLQ-C30, EQ5D-VAS) considered as measurements for disease symptoms in the German CA were considered as HRQoL instruments in the EU JCA. As the differing allocation did not affect the acceptance/non-acceptance of the respective endpoint, these differences were rated as having only minor impact. However, in line with the scope of the HTA, the consideration of patient-reported symptoms and HRQoL is advocated to enable a patient-centred assessment.

Any inclusion of additional information or data in the EU JCA compared to the German CA, that had no impact on the conclusion in the outcome categories of the relative effectiveness assessments was considered to have no direct impact on the compatibility of the CA.

### Definition of additional comparators, endpoints, subpopulations or sensitivity analyses in EU JCA

Our analysis showed that typically the EU JCA authors defined additional comparators to those included into the German CA. The German criteria for the selection of relevant comparators give preference to established and approved therapies in the indication of interest. To be eligible as comparators, off-label therapies require a positive benefit assessment by the off-label commission of the G-BA. Relevant comparators in the EU JCA do not have to fulfil the same criteria; the inclusion of off-label comparators is possible if needed to reflect national therapy standards. For future EU JCA, medicines with marketing authorisation for the therapeutic indication should be given priority, off-label therapies with demonstrated clinical efficacy for the therapeutic indication should be considered. The selection should be made according to available clinical evidence and European guidelines.

The definition and analyses of subpopulations additionally to label population, the inclusion of additional endpoints, sensitivity analyses or sources of evidence in the EU JCA scope have no direct impact on the transferability of EU JCA. Generally, subgroup analyses should be considered very cautiously due to their possible exploratory character. Conclusions about differential effects in subgroups should only be drawn based on adequate methodology.

### Additional indirect comparisons or additional results in the EU JCA

Typically, in the EU JCA, additional indirect comparisons were included. Regarding subgroup analyses, no trend was observed. In some examples, additional subgroups were analysed for the German CA, in others for the EU JCA, but typically without affecting the conclusions from the primary analysis.

In the EU JCA, all available evidence must be presented for all comparators, if necessary by indirect comparison. In the German context, on the other hand, if several comparators—which are perceived as equal—have been defined, it is possible for the sponsor to select one of them for evidence presentation. If no direct comparison for any of the comparators is available, indirect comparisons as best available evidence can and should be considered for German CA [23].

#### Limitations

An important limitation of our analysis is the inclusion of predominantly oncology products (3 out of 5), of which 2 are orphan medicinal products (Table 1). Therefore, results may not be generalizable to all indications. The scope of this comparison did not include the assessment of possible variability of JCA due to the involvement of different author countries. Moreover, no scale exists to classify the impact of differences as advantage or disadvantage. Overall, as this analysis comprises only a small sample size, the results should be interpreted with caution. Only JA3 EU CA were included in the analysis, limiting the available dataset. Earlier EU JCA still varied in scope, content and processes and are therefore not well comparable with each other. The focus on JA3 assessments allowed for the systematic comparison of EU JCA and German CA based on the latest EU JCA methodology. We applied a hierarchical approach which distinguishes our analysis from previously published comparisons assessments.

## Conclusion

To meet the goal of a reduction in submissions and assessments, future EU JCA should constitute a suitable basis for subsequent HTA decisions on added value in the specific national healthcare context. For this purpose, common standards reflecting the main requirements of the CA in the Member States are necessary and compromises have to be found for the definition of guidelines. Our analysis showed that there are already many synergies between EU JCA and German CA. However, we still identified several aspects in HTA methodology that would benefit of harmonisation to ensure the transferability of future EU JCA to the German HTA process without duplicated evaluation requirements, while meeting the overall goal of ensuring a fast and equal access by patients to medicines across Europe.

## Supplementary Information

Below is the link to the electronic supplementary material.

Supplementary file1 (DOCX 22 KB)

Supplementary file2 (DOCX 28 KB)

## Data Availability

Not applicable.
