# Supplementary material for: Towards compatibility of EUnetHTA JCA methodology and German HTA: a systematic comparison and recommendations from an industry perspective
Source: Eur J Health Econ. 2021 Nov 12;23(5):863–78. doi: 10.1007/s10198-021-01400-2 (PMC9170646; doi:10.1007/s10198-021-01400-2)
Supplement: Supplementary file 1 — Supplementary file1 (DOCX 22 KB) [file 10198_2021_1400_MOESM1_ESM.docx]

Supplementary table 1: Definition of assessment elements, and probing questions.

| ***A. Population*** | |
| --- | --- |
| A1 | What was defined as the relevant population for the (J)CA (scope)? (including subpopulations, if applicable) |
| A2 | Did the relevant population (according to project scope) correspond to the approved indication^1^? (Y/N/NA) |
| A3 | Were subpopulations defined? (Y/N/NA)^2^ |
| A4 | Were the study populations (of the pivotal studies) considered suitable for the REA? (Y/N/NA) |
| ***B. Intervention*** | |
| B1 | What was defined as relevant intervention for the (J)CA (scope)? |
| B2 | Did the relevant intervention (according to project scope) correspond the approved application^3^? (Y/N/NA) |
| B3 | Was the implementation of the intervention in the registration studies assessed as suitable for the REA? (Y/N/NA) |
| ***C. Comparator*** | |
| C1 | Which comparative therapies were defined as relevant for the (J)CA (scope)? |
| C2 | Were direct studies with the defined comparators available? (Y/N/NA) |
| C3 | Was the implementation of the comparative therapy in the direct comparative studies assessed as appropriate for the (J)CA? (Y/N/NA) |
| C4 | Were indirect comparisons taken into account? (Y/N/NA) |
| C5 | Were results from indirect comparisons used to derive the additional benefit? (Y/N/NA)^4^ |
| ***D. Outcomes (For each outcome category: mortality, morbidity, health related quality of life, safety)*** | |
| D1 | Which endpoints (in which operationalisation) were defined as relevant (scope)? |
| D2 | Was a conclusion on relative effectiveness/safety derived (advantage/no difference/disadvantage/NA)^4^? |
| D3 | How was the risk of bias ranked? (high/low/NA) |
| D4 | Was the reliability of the results in each category downgraded^4,5^? (Y/N/NA) |
| ***E. Subgroups*** | |
| E1 | Which subgroups were analysed? For which endpoints? |
| E2 | Did the results of the subgroup analyses influence the conclusion on relative effectiveness/safety? (Y/N/NA)^4^ |
| ***F. Sensitivity analysis*** | |
| F1 | Which sensitivity analyses were taken into account? For which endpoints? |
| F2 | Did the results of the sensitivity analyses influence the conclusion on relative effectiveness/safety? (Y/N/NA) ^4^ |
| ***G. Other sources of evidence*** | |
| G1 | Were other sources of evidence considered? (Y/N/NA) |
| G2 | Did the results of other evidence sources influence the conclusion on relative effectiveness/safety? (Y/N/NA) ^4^ |
| 1: According to SmPC section 4.1  2: If yes, list subpopulations and state if additional consideration has been given to the total population  3: According to SmPC section 4.1 and 4.2  4: According to the conclusion or summary of findings table (EU JCA) or summary table on additional benefit (German CA)  5: According to GRADE  N: No; NA: Not appicable; (J)CA: (joint) clinical assessment; Y: Yes. | |
