# Supplementary material for: Towards compatibility of EUnetHTA JCA methodology and German HTA: a systematic comparison and recommendations from an industry perspective
Source: Eur J Health Econ. 2021 Nov 12;23(5):863–78. doi: 10.1007/s10198-021-01400-2 (PMC9170646; doi:10.1007/s10198-021-01400-2)
Supplement: Supplementary file 2 — Supplementary file2 (DOCX 28 KB) [file 10198_2021_1400_MOESM2_ESM.docx]

Supplementary Table 2: Comparison of conclusions of EU JCA and German CA on endpoint category level^1^

|  | ***EU JCA conclusion*** | ***Comment*** | ***German CA***  ***conclusion*** | ***Comment*** |
| --- | --- | --- | --- | --- |
|  | ***Conclusion on Mortality*** | | |  |
| Alectinib | No difference | No statistically significant difference in overall survival. | No difference | "On overall survival, there is no evidence of reduced benefit or added benefit of alectinib." |
| Brolucizumab | NA | Mortality was not evaluated in the trial | NA | No relevant studies available. |
| Midostaurin | Advantage | “The risk of death was reduced by 23% during the follow-up for midostaurin versus placebo. The proportion of patients alive was significantly higher at the 1- and 5-year follow-ups, demonstrating both short and long-term positive effects of midostaurin on survival.” | Advantage | “There was a statistically significant reduction in mortality risk in the intervention group compared to the control group. The median survival benefit in months is not validly quantifiable.” |
| Polatuzumab | Advantage | “The data showed a difference to the advantage of polatuzumab + bendamustine and rituximab versus bendamustine and rituximab for the following endpoints: […], overall survival, […]” | Advantage | “Statistically significant advantage in overall survival in favour of polatuzumab+ bendamustine and rituximab.” |
| Siponimod | NA | “No results in the population of interest” | No difference | Additional benefit not proven. |
|  | ***Conclusion on Morbidity*** | | |  |
| Alectinib | Advantage | “Alectinib demonstrated a substantial and statistically significant increase in progression-free survival (HR 0.47 (0.34, 0.65); 0,0001) and is also associated with a statistically significant longer time to CNS progression (HR 0.16 (0.10, 0.28); 0,0001) compared to crizotinib.” | No difference | "It is unclear whether and to what extent these beneficial effects of alectinib reflect prevention or delay of symptoms associated with CNS metastases or side effects of comparator therapy." |
| Brolucizumab | No difference | non-inferiority for BCVA; no conclusions can be drawn for injection frequency due to different treatment schemes; measures of anatomic outcomes only presented as supporting evidence ("clinical relevance of observed differences can not be evaluated") | NA | No relevant studies available. |
| Midostaurin | NA | Morbidity results are not mentioned in the conclusion. | NA | Morbidity results are only included as supplementary information |
| Polatuzumab | Advantage | “The data showed a difference to the advantage of polatuzumab + bendamustine and rituximab versus bendamustine and rituximab for the following endpoints: complete response, […]and PFS.” | NA | “Complete response and the neuropathy questionnaire (TINAS) were not considered valid to infer an additional benefit”. |
| Siponimod | NA | “No results in the population of interest” | No difference | Additional benefit not proven. |
|  | ***Conclusion on HRQoL*** | | |  |
| Alectinib | No difference | “Patients receiving alectinib had clinically meaningful improvement in HRQoL for a longer duration compared with patients receiving crizotinib, but the difference was not statistically significant.” | No difference | Difference was not statistically significant. |
| Brolucizumab | NA | NEI VFQ-25 was collected - but ambiguities regarding operationalisation (better/worse eye) - no clinically meaningful results. | NA | No relevant studies available. |
| Midostaurin | NA | QoL was not evaluated in the trial | NA | QoL was not evaluated in the trial |
| Polatuzumab | NA | QoL was not evaluated in the trial | NA | QoL was not evaluated in the trial |
| Siponimod | NA | “No results in the population of interest” | NA | QoL was not evaluated in the trial |
|  | ***Conclusion on Safety*** | | |  |
| Alectinib | Advantage | "While conclusions on relative safety compared with ceritinib should be made with caution, both  the NMA and the comparison of the established adverse events profiles in the SmPCs indicate an overall superior safety profile of alectinib." | Advantage | "In the present assessment, the additional benefit is mainly based on a reduction in some side effects." |
| Brolucizumab | Disadvantage | “Incidence of intraocular inflammation and retinal artery occlusive events were higher for brolucizumab.” | NA | No relevant studies available. |
| Midostaurin | No difference | “Comparable safety profile.” | Disadvantage | “Disadvantage (CTCAE grade 3-4) exfoliative dermatitis and aid-associated infections. […]  No differences in overall rates. […]  However, overall disadvantage due to specific UE (dermatitis and HM-ass infections).” |
| Polatuzumab | No difference | “According to the analysis performed by IQWiG, the data available did not show a difference between polatuzumab + bendamustine and rituximab and bendamustine and rituximab for the following endpoints: serious adverse events, severe adverse events (CTCAE Grade 3-4), and discontinuation due to adverse events.” | Disadvantage | Significant disadvantage for UE/therapy discontinuation + AESI: Peripheral neuropathy; significant advantage Cardiac toxicity;  Selective choice of safety endpoints |
| Siponimod | NA | “No results in the population of interest” | NA | No usable evaluations |
| **^1^**The information presents the answers to question D2 from Supplementary Table 1 together with the instructions for answering from the evidence table. See Table 1 for respective relevant comparators in the (J)CA  NA Not applicable/available | | | | |
